# The enterprise’s external knowledge acquisition capability and technological diversification: From the perspective of intellectual property strategy

**DOI:** 10.3389/fpsyg.2022.1093362

**Published:** 2023-01-04

**Authors:** Ke-Chiun Chang, Yukun Jiang, Chengjing Huang, Xinyi Xiong, Ziyan Chen, Yen-Chun Lai, Kuang-Cheng Chai

**Affiliations:** ^1^School of Economics and Management, Wuhan University, Wuhan, China; ^2^College of Law, Zhongnan University of Economics and Law, Wuhan, China; ^3^Business School, Guilin University of Electronic Technology, Guilin, China

**Keywords:** absorption capacity, relationship learning, technological diversification, innovation ability, environmental uncertainty

## Abstract

Enterprises need intellectual property rights to protect their core knowledge, and technological diversification is an important strategic measure for enterprises to improve innovation performance. From the perspective of external resource acquisition, this study explores the mechanism of external knowledge acquisition capability (internal absorptive capability and external relational learning) on firm’s technological diversification. It considers the impact of firm’s innovation capability and external environmental uncertainty. The survey data of 258 Chinese pharmaceutical companies were obtained through questionnaire surveys, and various theoretical hypotheses were validated using regression analysis methods. The results show that internal absorptive capacity, external relational learning, and their interaction have a significant positive impact on technological diversification; the innovation capacity and the uncertainty of the external environment also affect enterprises’ technological diversification.

## 1. Introduction

Intellectual property is the exclusive right of intellectual capital. In open innovation mode, enterprises need more intellectual property protection systems to protect the core knowledge. Open innovation emphasizes the importance of external knowledge resources for enterprise innovation and holds that enterprises should purposefully let technology, knowledge, experience, and talent flow into accelerated innovation to obtain strategic competitive advantages (Chesbrough, 2003; Chesbrough et al., 2006). In other words, enterprises adopting technological diversification can further strengthen their technological innovation ability. In recent years, in the context of the transformation from factor-driven to innovation-driven, Chinese firms have shown a strong upward trend in creating new products and new industries with rapid learning, accumulation, and application of technology knowledge (Zhang et al., 2020), the overall innovation capacity of Chinese enterprises is still weak, such as the lack of high-level technological innovation achievements, the absence of basic research and the serious shortage of applied research. Exploring the driving factors of innovation has become a topic of common concern in practice and academic circles. As an important driving force of enterprise technology innovation, technological diversification is also a classic problem in innovation management research. Technological diversification was first defined by Kodama (1986) as an enterprise’s R&D activities outside the main product area. Patel and Pavitt (1997) further defined technological diversification as an activity in which enterprises extend their technical knowledge beyond the core technical fields. This study refers to Chen and Chang (2012) to define technological diversification as the extent to which a firm diversifies its technological capabilities in relevant or irrelevant technological fields. Specifically, it can be divided into two types; the first type is multiple utilization of technology, that is, an enterprise uses one technology in many products or services to rapidly promote new products or services to the market. Rely on technology’s scope and scale economy to obtain profits and increase the added value of existing products; the second type is for companies to expand their technological capabilities to a wide range of technical fields and acquire technological assets to form R&D diversification.

In previous studies on enterprise’s technological diversification, some scholars also studied the factors affecting technological diversification. Some literature analyzed the influencing factors of technological diversification based on the nature of knowledge, and the research level was relatively simple. For example, Breschi et al. (2003) pointed out that the key factor affecting technological diversification was knowledge relatedness. It uses three dimensions of knowledge proximity, commonality, and complementarity to measure the degree of knowledge correlation. Yao et al. (2020) pointed out that knowledge sharing can impact technological diversification. Chiu et al. (2008) subdivided the three major aspects: environmental, strategic orientation, and resource endowments. Then they pointed out that the higher the degree of environmental richness, industrial attraction, R&D intensity, and vertical integration, the higher the degree of enterprise’s technological diversification, and the higher the degree of industrial competition and reversible margin, the more specialized the enterprise’s technology will be. However, the influence of the interaction of factors at all levels on enterprise’s technological diversification remains a largely unexplored topic. This article seeks to contribute to bridging this gap in the literature by further investigating the influencing factors of technological diversification.

Under the development trend of enterprise technological innovation mode from closed to open gradually, whether an enterprise can acquire knowledge and technology from outside and convert it into its own knowledge through internal absorptive capacity has become the key to successful innovation (Albort-Morant et al., 2018; Flor et al., 2018). Access to external resources helps enterprises acquire heterogeneous knowledge from partners and research institutions (West et al., 2014), thus promoting technological diversification, adapting to changes in the external environment, and reducing the risk of technology lock-in. In this context, the technological diversification of enterprises depends not only on the digestion and accumulation of internal knowledge but also on the acquisition and learning of external knowledge. Absorptive capacity refers to the ability of an enterprise to identify potential external knowledge and to digest and use it (Cohen and Levinthal, 1989). Enterprises with strong absorptive capacity can obtain a large amount of valuable information from outside for their own use, which provides sufficient knowledge accumulation for the technological diversification of enterprises (Roberts et al., 2012; Flor et al., 2018). At the same time, literature on supply chain partnership has pointed out that information sharing and knowledge learning are important channels for enterprises to acquire explicit and even tacit knowledge (Dyer and Nobeoka, 2000; Najafi-Tavani et al., 2018). In addition, studies have found that when enterprises use the technology of external partners, the degree of technological diversification tends to increase (Krammer, 2016). Therefore, from the perspective of external resource acquisition, this article will study how the internal absorptive capacity and external relationship learning influence the willingness and degree of technological diversification of enterprises, so as to deepen the understanding of enterprises’ implementation of technological diversification strategy.

This article continues by offering a theoretical background on technological diversification and on two core abilities for enterprises to acquire external resources. We then develop our theory and specific predictions. In the following parts, we describe measures and proxies, econometric methods, and report results. In the end, we discuss these findings and conclude the article.

## 2. Literature review and hypothesis development

### 2.1. Ability to acquire external resources and technological diversification

As mentioned earlier, acquiring diverse technological knowledge is required for enterprises to achieve innovations beyond the existing core technical field and thus gain technological diversification. The relationship between the enterprise and the outside and its internal absorptive capacity are two key aspects of open innovation (Xia and Roper, 2016; Albort-Morant et al., 2018). Relationships are the channels for enterprises to acquire external knowledge; the more extensive the enterprise’s relationship network is, the greater the scope of external knowledge acquisition will be (Najafi-Tavani et al., 2018). Different types of relationships lead to differences in acquired knowledge; for example, it is easier to acquire combinational knowledge in direct relationships, while it is easier to acquire heterogeneous new knowledge in indirect relationships (Singh et al., 2016). In addition, the establishment and maintenance of the relationship also need to weigh the cost-effective. Although the establishment of a technology alliance for the purpose of R&D can bring high benefits to enterprises, the maintenance costs are correspondingly high, which is difficult for enterprises with small scale and weak R&D capacity to implement. The upstream and downstream partnership formed based on business transactions is characterized by “inherency” and “flexibility.” Enterprises can consciously strengthen mutual trust and promote information sharing and learning exchange on the basis of business transactions. In this process, how much knowledge an enterprise obtains from the outside and whether the knowledge it obtains is what it needs depends on the strength of its relationship learning ability (Najafi-Tavani et al., 2018).

Moreover, acquiring external resources through internal absorptive capacity is a consistent view in the open innovation research literature (Grant, 1996; Chesbrough, 2003; Chesbrough et al., 2006; Xia and Roper, 2016). Absorptive capacity emphasizes the recognition, acquisition, transformation, and utilization of external knowledge and is the integration and accumulation of knowledge. With the increase of the depth of knowledge, the enhancement of absorption capacity, and the improvement of the ability to identify external information, enterprises will acquire more valuable external knowledge. The main purpose of this study is to discuss which specific capabilities can help enterprises acquire external knowledge, update their own knowledge accumulation and maintain continuous technological diversification from the perspective of external resource acquisition. To sum up, this article divides external resource acquisition ability into external relationship learning ability and internal absorption ability.

### 2.2. Internal absorption capacity and technological diversification

Cohen and Levinthal (1990) first proposed the concept of absorptive capacity and defined it as the ability of an enterprise to value, absorb, and utilize external knowledge; the extent of such capability largely depends on the relevance of knowledge among enterprises (Lane and Michael, 1998). On this basis, Zahra and George (2002) further expanded the concept and divided absorptive capacity into two stages: potential absorptive capacity and actual absorptive capacity. The former is the collection of acquisition and digestion capacity, emphasizing the ability of enterprises to acquire new knowledge from the outside and understand and internalize it. The latter is a collection of transformation and utilization capabilities, emphasizing the integration of new knowledge and optimization of original technical knowledge to improve the utilization rate of knowledge. Meanwhile, Albort-Morant et al. (2018) argued that managers should devote more time and resources to reinforce their absorptive capacity as an important strategic tool to generate new knowledge. Therefore, this article believes that the absorptive capacity of an enterprise is composed of two capabilities in different stages of its knowledge processing, namely potential absorptive capacity and actual absorptive capacity, which affect technological diversification from different stages and aspects, respectively.

From the perspective of potential absorptive capacity, the stronger the potential absorptive capacity is, the more likely an enterprise is to search for and introduce valuable external information, increase the possibility of combining with its own knowledge, and provide the necessary knowledge base for enterprise technological diversification. In addition, the development of technology R&D guided by diversified market needs is more conducive to the commercialization of technology. Enterprise with strong potential absorptive capacity is more sensitive to external knowledge and has a more accurate grasp of market demand; therefore, in order to obtain leader advantages and avoid technology lock-in, such companies are more motivated to increase R&D intensity and implement technological diversification. The knowledge that an enterprise absorbs from the outside world is valuable knowledge only when it is truly digested and utilized by itself. The strength of actual absorption capacity determines the degree of transformation and utilization of external knowledge. The transformation and utilization of new knowledge can promote the integration and upgrading of new and old knowledge and improve the possibility of knowledge spillover. By integrating new and old knowledge, brand-new knowledge will be formed, increasing the difference between technologies and improving the degree of technological diversification.

The two stages of absorptive capacity both affect technological diversification, but it does not mean that they independently affect technological diversification; in fact, it is a continuous process. Potential absorptive capacity by identifying external valuable knowledge and introducing it into the enterprise, through the transformation and utilization of the real absorptive capacity, the enterprise’s own knowledge reserve is formed, and the transformation effect will be feedback to the potential absorptive capacity, so as to further optimize the enterprise’s ability to identify and acquire external resources. Therefore, enterprises with stronger absorptive capacity are more capable and willing to diversify their technologies. Therefore, this article proposes the following hypothesis:

*H1: Internal absorptive capacity has a significant positive impact on the level of technological diversification.*

### 2.3. External relationship learning and technological diversification

Acquiring knowledge and resources by learning from partners is the focus of inter-organizational learning and knowledge creation (Zander and Kogut, 1995; Hitt et al., 2000). Relationship learning is a kind of organizational learning, which has the characteristics of organizational learning. However, relationship learning involves partnership; the relationship between enterprises and different partners is different, and at the same time, the importance of the partnership of enterprises also changes with different partners, which gives relationship learning its own uniqueness (Lukas et al., 1996; Selnes and Sallis, 2003). First, the relationship memory of both partners is relative and different from organizational memory, and the relationship memory of different enterprises is different. Relationship learning includes the common history, reference structure, and values of both organizations. Because memories are shared, they are the public resource for both partners to use regardless of the status of the partners. Second, the conditions of relationship learning are different from those of organizational learning, relationship learning requires joint efforts of both parties, and relationship learning cannot be carried out with only a one-sided willingness to learn. In addition, relationship learning will bring different results to the organization than organizational learning. At present, there is no conclusion about the results of relationship learning and organizational learning in the academic circle. A mainstream understanding is that the influence objects of relationship learning and organizational learning are different, and relationship is affected by relationship learning, and organization is affected by organizational learning. Therefore, relationship learning attaches great importance to relationship-building ability (Hallén et al., 1991). What kind of relationship to establish will directly affect the effectiveness of learning?

Starting from the relationship between the enterprise and upstream suppliers and downstream customers, this article believes that relationship learning is composed of information sharing, mutual understanding, relationship-specific information, and memory integration. Specifically, it refers to uninterrupted participation in the process common activities between customers and suppliers, sharing information, participating in the formation of perceptions and developing special relationships, and performing specifically related memory improvement and potential specific relationship behaviors, after a series of systematic integration, and then publicly sharing information (Selnes and Sallis, 2003). Learning depends on the willingness of both parties to cooperate in the process of relationship learning activities. Cultivating cooperative cultural management can promote relationship learning, articulate specific goals for learning activities, and develop relevant trust. They also find that relationship learning ability is strong and has a positive impact on performance.

With the drastic changes in the competitive environment and the acceleration of updating technical knowledge, learning for enterprises is not only limited to the internal of enterprises but also needs to be extended and infiltrated among organizations. The relationship provides a necessary way for enterprises to obtain external knowledge. Research points out that a relationship is the “pipe” through which knowledge flows between different individuals, and the type of relationship affects the type of knowledge acquired by individuals (Singh et al., 2016). Therefore, through relational learning, enterprises can absorb the operation experience of relational enterprises, acquire knowledge and resources that are beneficial to them, understand the professional technical knowledge in the market more deeply, and use this to guide the layout strategy of enterprises in various technical fields, and reduce the cost and risk of technology modeling. Moreover, Breschi et al. (2003) proposed that the correlation of knowledge is an important factor affecting the technological diversification of enterprises, and enterprises with a higher degree of diversification have higher internal consistency of knowledge. Because the enterprises with upstream and downstream supplier relationships have the characteristics of target consistency, business complementarity, and knowledge heterogeneity, the knowledge acquired through this relationship is more relevant to the target enterprises, and the enterprises are more likely to carry out technological diversification. Therefore, this article proposes the following hypothesis:

*H2: External relationship learning has a significant positive impact on the technological diversification of enterprises.*

### 2.4. The interaction between internal absorptive capacity and external relational learning

Cohen and Levinthal (1990) pointed out that there was a bidirectional relationship between absorptive capacity and organizational learning; organizational reinforcement learning in a certain field would enhance the absorptive capacity of the organization; conversely, the enhancement of absorptive capacity will promote the learning of the organization in this field. Relational learning is a kind of organizational learning; combined with the research scenario of this article, it mainly emphasizes the process of core enterprises acquiring, sharing, interpreting, and remembering new knowledge from the cooperation with upstream and downstream suppliers. Usually, the upstream and downstream partners of a company have better knowledge accumulation in their own fields; the stronger the company’s relationship learning ability, the more valuable new knowledge or even a combination of knowledge from them and enhance their own understanding and cognition of a certain field at a lower cost, thereby improving the enterprise’s ability to identify and acquire external knowledge. In addition, relationships are also the “pipeline” of knowledge flow between different individuals, rather than searching for knowledge from an infinite resource pool, enterprises should acquire knowledge more effectively through relationships (Singh et al., 2016). Therefore, from the perspective of social network theory, the relationship between an enterprise and its upstream and downstream suppliers is also the social capital owned by the enterprise, and the value of such social capital is also affected by the internal capabilities of the enterprise (Lee et al., 2001). The influence of absorptive capacity on relationship learning is reflected in two aspects: First, enterprises with strong absorptive capacity will consciously maintain the partnership, enhance mutual trust, and expand information exchange and knowledge sharing on this basis due to their dependence on external knowledge acquisition; second, the enterprise behavior has the profit-driven, the diversified knowledge acquired through relational learning can only be intended to implement by the enterprise when it can serve the product or process, and the stronger the absorption ability, means that the higher the efficiency of the enterprise’s conversion and utilization of the learned knowledge, which will help the company to expand in a certain technical field or extend to multiple technical fields, and affect the diversification of technology. In conclusion, based on the symmetry of this interaction, this article believes that external relationship learning can promote the technological diversification of enterprises, and technological diversification will be further improved with the enhancement of internal absorption capacity. Therefore, this article proposes the following hypothesis:

*H3: The influence of internal absorptive capacity and external relational learning on technological diversification is mutually promoting, that is, enterprises with both capabilities will show a higher degree of technological diversification.*

## 3. Methodology and measurement

### 3.1. Sample and data collection

The selection criteria of research objects in this study are as follows: pharmaceutical enterprises with continuous R&D investment, continuous product and process innovation activities, and relatively frequent technical activities. At the same time, the establishment time of sample enterprises is required to be at least 5 years, to ensure that they have enough time to build up their own knowledge and technology reserves, have a certain degree of absorption capacity and relationship learning ability, and have a clear range of their own technical knowledge. The sample companies in this article were chosen for the following considerations: although these enterprises are widely distributed in scale, are in different industries, and have different product and technology development strategies, their foothold in the industry and obtaining competitive advantages must be highly dependent on technological innovation, which fits well with the context of this research. Therefore, the research object of this study is the biological pharmaceutical enterprises that have been established for more than 5 years in China and have carried out certain product or technological innovation activities.

This study collected data through questionnaires. The middle and senior managers and technical leaders of enterprises play a leading and decision-making role in the technical activities of enterprises. They constantly plan and implement the technical development plan of the enterprise and can better grasp the technical situation of the enterprise and the overall status of the enterprise’s performance. Selecting appropriate respondents can improve the authenticity and accuracy of data. Therefore, the respondents of this questionnaire are all middle and senior managers and technical leaders of enterprises. In addition, all variables were measured using a 5-point Likert scale, with 1 to 5 representing strongly disagree to strongly agree. The collected data were finally statistically analyzed using SPSS.

During February–March 2021, questionnaires were distributed and collected in this survey in two ways: (1) On-site questionnaires were distributed to MAB and EMBA class students at the Wuhan university, a total of 60 questionnaires were distributed and 50 were recovered, of which 35 were valid. The effective questionnaire recovery rate was 70%. (2) Sending questionnaires to the target enterprises by email to ensure that each enterprise sends only one questionnaire. A total of 300 questionnaires were issued, and 273 were recovered, among which 223 were valid. The effective questionnaire recovery rate was 81.68%.

Finally, 258 valid questionnaires were obtained, which constituted the sample of this study. The regional distribution of the final sample enterprises is shown in Table 1.

**TABLE 1:** Regional distribution of sample enterprises.

Regions	Quantity	Percentage (%)
Guangdong	56	21.70
Shanghai	32	12.40
Beijing	28	10.85
Hubei	35	13.56
Fujian	17	6.58
Zhejiang	16	6.20
Jiangsu	24	9.30
Sichuan	13	5.03
Shandong	17	6.58
Liaoning	20	7.75
Total	258	100

### 3.2. Measurement

The measurement of technological diversification is a key issue in empirical studies, but there is no unified solution to measure it (Granstrand and Oskarsson, 1994; Chiu et al., 2008). In this article, the questionnaire survey method is used to let the middle and senior managers who are familiar with the overall technology of the enterprise give their self-evaluation of the technology situation of the enterprise, which can more accurately measure the level of technological diversification of an enterprise and improve the response rate of the questionnaire. This article refers to the scale of He (2011) for measurement.

Internal absorption capacity. This study refers to the scale of Cohen and Levinthal (1990); the reliability of the items in the scale has been verified in subsequent studies by scholars and can well measure the absorptive capacity of enterprises. Combined with this study, seven measurement items of absorption capacity were determined.

External relationship learning. Relational learning means that companies are able to develop common areas of learning through the exchange of information and optimize their initiatives according to the needs of their suppliers, customers, and partners. The research results of Selnes and Sallis (2003) laid a foundation for the subsequent study of relational learning theory, and the scale used by Selnes and Sallis is widely used. This study also referred to the classical scale, combined with the actual situation of this study, finally determined eight measurement items.

Control variables. Innovation ability is the basic ability for an enterprise to effectively carry out technological R&D activities, which directly affects the efficiency of its technological innovation activities, while technological diversification refers to the achievement of technological innovation activities in multiple fields, so an enterprise’s innovation ability will also have an impact on its technological diversification. However, since this article mainly studies the influencing factors of technological diversification from the perspective of external resource acquisition, innovation capability is taken as a control variable. The scale reference of enterprise innovation capability (Yalcinkaya et al., 2007).

Environmental uncertainty. Knowledge acquired from the outside will inevitably be affected by environmental uncertainty, so it is considered a control variable; the design of related items refers to the measurement methods of Kumar and Seth (1998).

### 3.3. Reliability and validity test

Cronbach’s α coefficients of the constructs are shown in Table 2. Generally, the minimum requirement of Cronbach’s α coefficient is 0.7. It can be observed that Cronbach’s α coefficient of “technological diversification” is 0.793; Cronbach’s α coefficient of “relationship learning” is 0.724; Cronbach’s α coefficient of “absorptive capacity” is 0.785; Cronbach’s α coefficient of innovation ability is 0.830; Cronbach’s α coefficient of “environmental uncertainty” is 0.750. Cronbach’s α coefficients of all four constructs are greater than 0.7. Therefore, this study is acceptable in reliability. At the same time, confirmatory analysis was conducted on the variables, and the results showed that the factor loading of each variable was greater than 0.5, indicating that each variable had good aggregation validity.

**TABLE 2:** Construct measurement, reliability, and validity.

Constructs	Number of items	Cronbach’s α	Remark
Technological diversification	7	0.793	Acceptable
Relationship learning	8	0.724	Acceptable
Absorptive capacity	7	0.785	Acceptable
Innovation ability	8	0.830	Acceptable
Environmental uncertainty	6	0.750	Acceptable

## 4. Results

The descriptive statistics of each variable are shown in Table 3. Both internal absorptive capacity and external relational learning positively correlate with technological diversification.

**TABLE 3:** Descriptive statistics and correlations.

Variables	Mean	S. D.	1.	2.	3.	4.
(1). Technological diversification	3.832	0.576	1			
(2). Relationship learning	3.989	0.451	0.624**	1		
(3). Absorptive capacity	3.951	0.549	0.738**	0.678**	1	
(4). Innovation ability	3.950	0.562	0.764**	0.674**	0.785**	1
(5). Environmental uncertainty	3.527	0.665	0.375**	0.256**	0.297**	0.264**

**p < 0.01.

This article adopts the regression method to verify the hypothesis, and the results are shown in Table 4. Model 1 shows the driving effect of control variables on the technological diversification of enterprises. According to the regression results, innovation capability and external environment uncertainty both have a significantly positive impact on the technological diversification of enterprises, that is, the stronger the innovation capability of enterprises or the greater the uncertainty of the external environment, the more inclined the enterprises are to carry out a technology diversification strategy.

**TABLE 4:** Regression results.

Variables	Model 1	Model 2	Model 3
Intercept	0.547** (2.767)	0.023 (0.104)	−0.209 (−0.884)
Absorptive capacity		0.267** (3.730)	0.309** (4.237)
Relationship learning		0.163* (2.305)	0.180* (2.558)
Absorptive capacity × Relationship learning			0.184* (2.408)
Innovation ability	0.716** (16.427)	0.440** (6.678)	0.430** (6.569)
Environmental uncertainty	0.145** (3.919)	0.116** (3.262)	0.121** (3.425)
Adj-R^2^	0.606	0.645	0.652
*F*-value	29.183**	30.167**	29.299**

**p < 0.01, *p < 0.05.

Model 2 shows the influences of internal absorptive capacity and external relational learning capacity on the technological diversification of enterprises. The regression results show that absorptive capacity and relational learning both have a significant positive impact on the technological diversification of enterprises; H1 and H2 are established, that is, starting from the acquisition of external resources, enterprises actively improve the ability to identify, acquiring, transforming and utilizing external resources will help enterprises to implement technological diversification and improve innovation performance. In addition, by learning from upstream and downstream partners, enterprises can also bring complementary resources to improve technological diversification.

On the basis of model 3, the effect of the interaction between absorptive capacity and relational learning on technological diversification was further verified. The regression results are significant, indicating that internal absorptive capacity and external relational learning influence each other. Firms with high absorptive capacity and relational learning capacity will show a higher level of technological diversification than those that have only one or a lower level of both.

## 5. Conclusion and discussion

Multiple factors that contribute to enterprises’ technological diversification have been identified, ranging from knowledge-relatedness to environmental aspects, strategic orientation, and resource endowments. The influence of the interaction of factors at all levels on enterprise’s technological diversification, however, remains a largely unexplored topic. This article seeks to contribute to bridging this gap in the literature. Specifically speaking, this article discusses the two key capabilities of external resource acquisition under open innovation-internal absorptive capability and external relationship learning capability, and the mechanism of the degree of technological diversification and willingness of enterprises. From the perspective of internal capacity, enterprises with stronger absorptive capacity have higher willingness and degree of technological diversification because high absorptive capacity means that enterprises can effectively acquire external knowledge and convert it into their own knowledge accumulation, laying a foundation for the implementation of technological diversification strategy. From the perspective of external capabilities, relational learning also has a significant positive effect on technological diversification, indicating that establishing a good learning relationship with upstream and downstream partners enhances trust between each other and helps enterprises obtain complementary and differentiated knowledge resources, thus avoiding single and rigid technology. Therefore, absorptive capacity and relational learning are both important capability factors affecting the technological diversification of enterprises, and these two capabilities complement each other and act on the whole process of technological diversification.

Implementation of technology diversification helps companies gain a competitive advantage; the previous literature also pointed out that capabilities and resources are the sources for companies to obtain sustainable competitive advantages, so this article research helps to open the black box from resources and capabilities to competitive advantages, revealing that in terms of external resource acquisition, enterprises improve their internal absorptive capacity and external relationship learning capacity at the same time, which can significantly promote technological diversification, accordingly enhancing their market competitiveness.

Therefore, in the aspect of cultivating the ability of technological diversification, enterprises should improve the absorptive ability and relational learning ability at the same time and promote technological diversification by complementing each other. To be specific, enterprise managers should consider improving the absorptive capacity of enterprises, establishing an open learning atmosphere for innovation, seeking relevant knowledge actively, learning and absorbing new technical knowledge from the outside efficiently, and internalizing it into the knowledge and technology reserve of enterprises. At the same time, enterprises should also strengthen the information flow with upstream and downstream partners, establish relationships with important positions organizations actively, especially informal relationships, obtain useful information from these information sources timely, constantly drive the technological diversification of enterprises and timely adjust development strategies. In addition, the research found that improving innovation ability also contributes to promoting technological diversification. The greater the uncertainty of the external environment, the more enterprises will maintain technological diversification. In a word, the technological diversification of an enterprise cannot be completed overnight; its formation and development cannot be separated from long-term and complicated work, and it has evolution and path dependence; it will involve various internal and external factors of the enterprise, and it has risks. Therefore, this article argues that it is of great guiding significance for enterprises to reduce trial and error costs and effectively implement a technology diversification strategy.

## Data availability statement

The raw data supporting the conclusions of this article will be made available by the authors, without undue reservation.

## Author contributions

K-CChan: project administration, conceptualization, and supervision. YJ, CH, XX, and ZC: investigation and validation. K-CChai: formal analysis. All authors designed and conducted the study, analyzed the data, wrote the draft, contributed to the interpretation of results, critically reviewed draft, and approved the final manuscript.
